# Practicable methods for histological section thickness measurement in quantitative stereological analyses

**DOI:** 10.1371/journal.pone.0192879

**Published:** 2018-02-14

**Authors:** Cyrill Matenaers, Bastian Popper, Alexandra Rieger, Rüdiger Wanke, Andreas Blutke

**Affiliations:** 1 Institute for Veterinary Pathology at the Center for Clinical Veterinary Medicine, Ludwig-Maximilians-Universität München, Munich, Germany; 2 Department of Anatomy and Cell Biology, Biomedical Center (BMC), Medical Faculty, Ludwig-Maximilians-Universität München, Martinsried, Germany; University of Campinas, BRAZIL

## Abstract

The accuracy of quantitative stereological analysis tools such as the (physical) disector method substantially depends on the precise determination of the thickness of the analyzed histological sections. One conventional method for measurement of histological section thickness is to re-embed the section of interest vertically to its original section plane. The section thickness is then measured in a subsequently prepared histological section of this orthogonally re-embedded sample. However, the orthogonal re-embedding (ORE) technique is quite work- and time-intensive and may produce inaccurate section thickness measurement values due to unintentional slightly oblique (non-orthogonal) positioning of the re-embedded sample-section. Here, an improved ORE method is presented, allowing for determination of the factual section plane angle of the re-embedded section, and correction of measured section thickness values for oblique (non-orthogonal) sectioning. For this, the analyzed section is mounted flat on a foil of known thickness (calibration foil) and both the section and the calibration foil are then vertically (re-)embedded. The section angle of the re-embedded section is then calculated from the deviation of the measured section thickness of the calibration foil and its factual thickness, using basic geometry. To find a practicable, fast, and accurate alternative to ORE, the suitability of spectral reflectance (SR) measurement for determination of plastic section thicknesses was evaluated. Using a commercially available optical reflectometer (F20, Filmetrics^®^, USA), the thicknesses of 0.5 μm thick semi-thin Epon (glycid ether)-sections and of 1–3 μm thick plastic sections (glycolmethacrylate/ methylmethacrylate, GMA/MMA), as regularly used in physical disector analyses, could precisely be measured within few seconds. Compared to the measured section thicknesses determined by ORE, SR measures displayed less than 1% deviation. Our results prove the applicability of SR to efficiently provide accurate section thickness measurements as a prerequisite for reliable estimates of dependent quantitative stereological parameters.

## Introduction

The thickness of a histological section generally affects the contrast, sharpness, and detail recognizability within the microscopic image of the slide. However, knowledge of the exact physical thickness of the examined section is not necessary in most qualitative histological analyses. Here, a certain degree of inter-section thickness variation is also acceptable, as long as an adequate quality of the section is maintained, allowing for a sound assessability of morphological parameters of the investigated tissue.

In contrast to such primarily qualitative histological analyses, application of distinct quantitative stereological analysis methods such as the physical disector, used for estimation of numerical volume densities (e.g. the number of cells per volume of tissue), fundamentally depends on a precise determination of the thicknesses of the analyzed histological sections.

### Physical disector analyses

**Fig 1:**
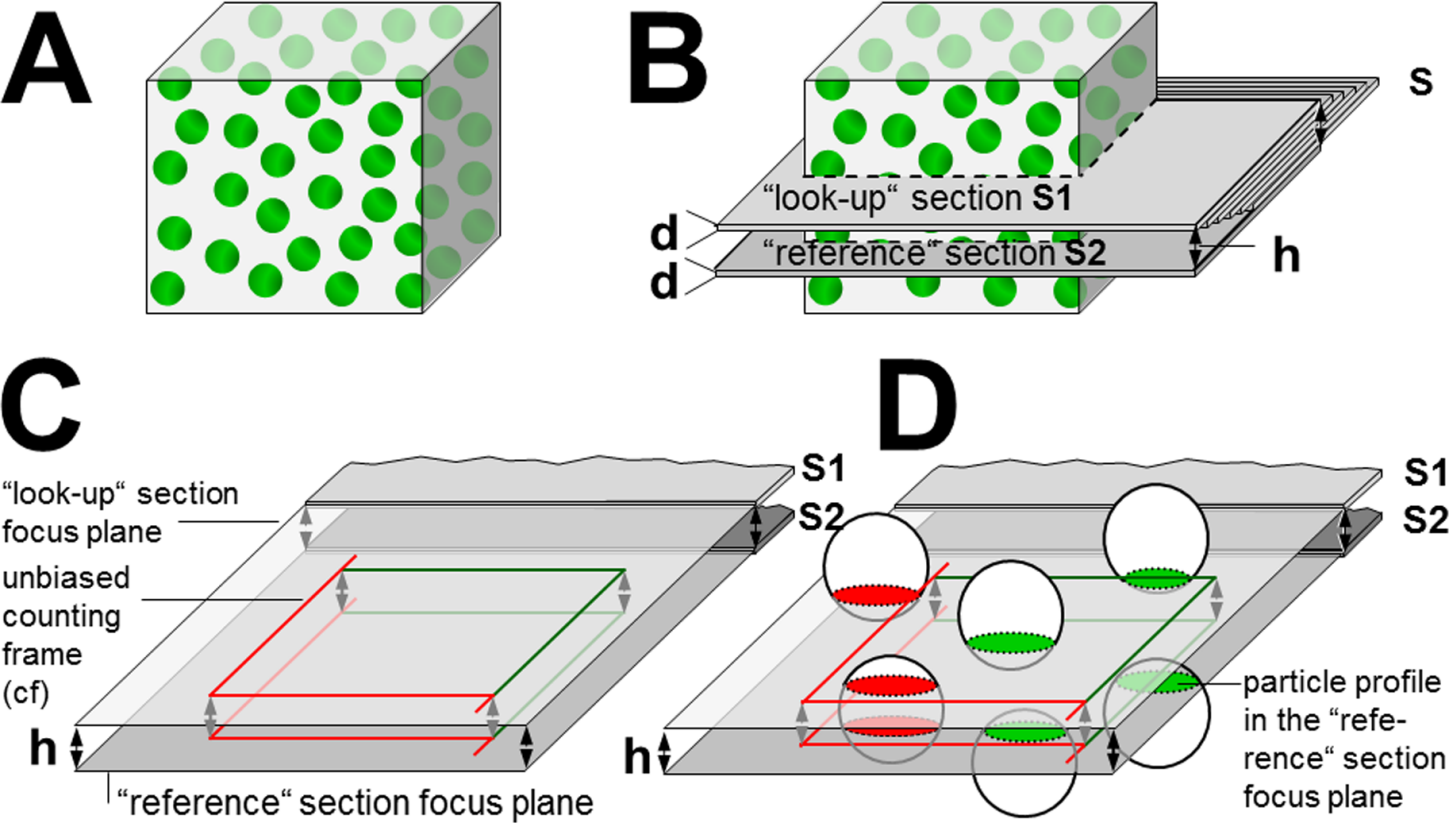
Schematic illustration of the physical disector method for quantitative stereological estimation of numerical volume densities. In this example, the numerical volume density of particles (green spheres) within a reference compartment (grey cube) is to be estimated (A). For a simplified presentation of the physical disector principle, the particles to be counted are equally sized spheres, evenly distributed within their reference compartment. A stack of parallel, equally thick sections (s) is cut from the reference compartment containing the particles of interest (B). The thickness of each of these sections is “d”. Two sections (a “look-up” section S1, and a “reference” section S2) are sampled with a known distance (h) between S1 and S2 (to avoid to miss particles completely located between the S1 and the S2 section, a disector height h of approximately ^1^/_3_^rd^ of the mean particle height perpendicular to the S1-S2 section planes is chosen). The distance h between the “look-up” section S1, and the reference section S2 is equal to the product of the number of sections between S1 and S2 +1 and the mean thickness (d) of these sections (here: h = 6d). C: A physical disector is a 3-dimensional test-system (probe) of known volume used for direct and unbiased counting of particles. Within the aligned, congruent (2D) focus planes of the “reference” and the “look-up” section, each one area is defined, in which particles being hit by either the reference section and/or the look-up section are sampled for counting. Here, an unbiased counting frame (cf) [2, 3] with “allowed” (green) and “forbidden” (red) lines is used. The disector volume in which the particles are counted is defined by the area of the counting frame (A_cf_) and the distance between the focus planes of the “reference” and the “look-up” section (i.e., disector height, h). D: Using the unbiased counting frame, particles are sampled for counting, if their section profiles in the reference section are either entirely within the counting frame or if they touch an “allowed”line but none of the “forbidden”lines of the counting frame. Particles whose section profiles hit one of the “forbidden”lines of the counting frame in the reference section are excluded from the analysis. Only sampled particles that hit the reference section but are not present in the “look-up”section are counted (sampled particles sectioned by the “reference“- and the “look-up”section are not counted). The process of counting might then be repeated with interchanged roles of the “reference“- and the “look-up”section, thereby doubling the effectiveness of the counting procedure. Thus, in the present example, four particles (green section profiles in the “reference“- or the “look-up”section) are counted in a corresponding reference compartment volume of two disector volumes (2 x h x A_cf_).

In disector analyses, structures of interest (e.g. cells or cell nuclei) are counted within defined volumes of tissue. The three-dimensional volume of a disector probe is defined by the distance (i.e., disector height) between two parallel section planes in the tissue (“reference”section and “look-up” section), and the 2-dimensional area within these section planes (e.g., an unbiased counting frame), in which the structures of interest are counted [1–3]. In a physical disector, the examined section planes are two (physically separate) histological sections taken from a series of consecutive, parallel, equally thick sections cut through the tissue (Fig 1). With regard to the potential anisotropy of the structure(s) of interest and depending on the size of the examined tissue sample, the position of the tissue sample relative to the orientation of the reference- and look-up section plane is randomized to generate isotropic uniform random (IUR) or vertical uniform random (VUR) section planes, using appropriate stereological designs [4–8]. The disector height h (i.e., the number of sections of known thickness between the reference section and the look-up section) is selected depending on the size of the counted particles. To avoid overlooking particles completely located between the reference- and look-up section, a disector height smaller than the mean particle height perpendicular to the section planes of the reference- and look-up section has to be chosen (usually approximately ^1^/_3_^rd^ of the linear orthogonal projection of the counted particles) [3, 6]. Therefore, if tiny structures such as small cell nuclei of 2–3 μm diameter are counted in a physical disector analysis, also comparably thin sections of 0.5–1.0 μm thickness (i.e., thinner than the mean minimal diameter of the particles counted) have to be applied, and, if appropriate, directly consecutive sections are used as the reference- and look-up sections. Knowledge of the disector height therefore depends on the knowledge of the exact thicknesses of the examined sections. The mathematical formula (Eq 1, Table 1) according to which numerical volume densities are calculated in physical disector analyses emphasizes the strong impact of the section thickness [1–3].

Eq 1

$${\hat{N}}_{V(X/Y)}=\frac{\sum_{i=1}^{n}Q^{-}{}_{{\left(X\right)}_{i}}}{h\sum_{i=1}^{n}A_{(Y)i}}$$ (1)

**Table 1:** Legend to Eq 1.

${\hat{N}}_{V(X/Y)}$	Numerical volume density of elements of the structure X in the reference compartment Y
$\sum_{i=1}^{n}{Q^{-}}_{{\left(X\right)}_{i}}$	Cumulative number of all counted elements (Q^-^) of the structure X in all disectors
$\sum_{i=1}^{n}A_{(Y)i}$	Cumulative area of the examined reference compartment sections in all disectors
h	Disector height (distance between the focus planes of the “reference”- and the “look-up” section)
n	Number of disectors analyzed per case
$h\sum_{i=1}^{n}A_{(Y)i}$	Cumulative volume of all disectors analyzed per case

It is obvious that the numerical volume density of any examined structure within its respective reference compartment does not only depend on the counted numbers of distinct elements of the structure of interest, but, at least equally as important, also on the volume of the reference compartment in which the structures were counted. Although, it is astonishing that in many published studies applying quantitative stereological physical disector analyses, determination of section thicknesses is not performed. Instead, the nominal section thicknesses set at the microtome are used for calculation of disector heights without verification. However, with regard to the considerable error effect of incorrect section thicknesses on the accuracy of the numerical volume density estimates obtained by physical disector analyses, it is negligent to refrain from determining the thicknesses of the examined sections [9]. In this context, it is irrelevant, if the true section thicknesses are actually identical with the nominal section thickness preset at the microtome, or not, because adoption of unnecessarily not verified assumptions on the thicknesses of the examined sections is hardly compatible with generally accepted standards of good laboratory practice. Actually, there is a considerable number of factors, which might actually result in deviations of the factual from the nominal thicknesses of histological sections. These factors include e.g., the brand, quality, manufacturing standards, age, utilization rate, operational performance and service intervals of the microtome, the individual operator, the hardness of the embedding medium, the time the tissue-block was stored before sectioning, the temperature during sectioning, and the type and condition of the microtome blade.

In this context, it should not left unmentioned, that there are structure analysis approaches [10], as well as several quantitative stereological analysis techniques [6], including methods for estimation of numerical volume densities, which are independent of section thicknesses or embedding-related tissue shrinkage. The latter procedures include several derivatives of the “fractionator” sampling method [9] combined with physical disector analyses [2, 6, 11]. However, in the experimental design of a study, it may be difficult to integrate the demands of “fractionator”- sampling regimes and those of other additionally scheduled analyses (e.g., generation of tissue samples for molecular-biological analyses), especially, if the available amounts of sample materials are limited. Therefore, section thickness-depending physical disector analyses are often given preference.

### Advantages of plastic embedding media in physical disector analyses

**Fig 2:**
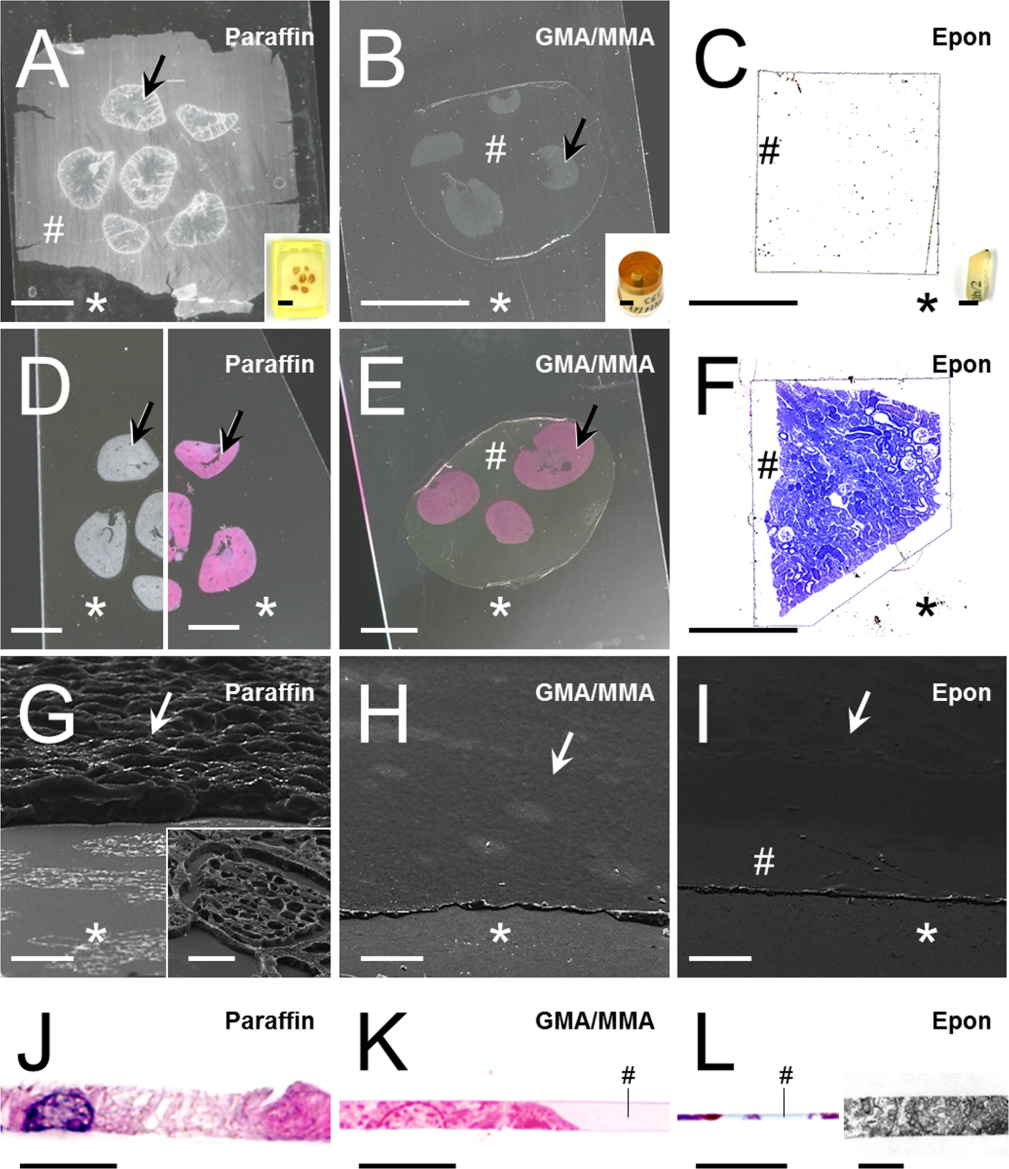
Morphology of paraffin- and plastic-sections. **A-I:** Perspective view on the relief of sections of paraffin sections and GMA/MMA- or Epon- plastic sections containing murine kidney tissue. Section areas with profiles of embedded tissue are indicated by arrows, section areas containing embedding medium without tissue are indicated by hashes (#), and the surface of the glass slides the sections are mounted on are indicated by asterisks (*). A-C. Freshly cut paraffin- (A), GMA/MMA- (B) and Epon- (C) sections mounted on glass slides after stretching of the sections on warm water-baths. Insets show paraffin, GMA/MMA- and Epon-blocks. D-F: HE-stained paraffin- (D) and GMA/MMA-sections (E) and Toloidine-blue-stained Epon- (F) section prior to mounting of cover-slips. D: Note that in paraffin-sections, the paraffin is removed during the processing of the section. Left image: Deparaffinized section prior to staining. Right image: HE-stained paraffin section. E, F: In plastic sections, the embedding medium (#) remains present in the section. Bars in A, B, inset to C, D and E = 5 mm. Bars in C and F = 0.5 mm. G-I: Scanning electron microscopic images of paraffin- (G), GMA/MMA- (H) and Epon- (I) sections. Note the uneven section surface and the absence of embedding medium in the paraffin-section, as compared to plastic sections. In both GMA/MMA- and in Epon-sections, the section surfaces of areas with and without embedded tissue are evenly smooth and at the same level. Bars = 20 μm. J-L: Orthogonal sections of paraffin- (J), GMA/MMA- (K) and Epon- (L) sections. J, K, L (left image): Light microscopic images. L (right image): Transmission electron microscopic image. Bars = 5 μm. Note the uneven surface of the paraffin-section, and the even level of the surface of plastic-sections in areas with and without (#) embedded tissue.

Physical disector analyses usually use sections of plastic resin-embedded tissue samples such as GMA/MMA (glycolmethacrylate/methylmethacrylate) [6] or Epon (diglycid ether 100), because plastic embedding causes less, and more uniform embedding-related tissue shrinkage than paraffin-embedding [6]. The extent of embedding related tissue shrinkage, usually expressed as the linear tissue shrinkage factor (f_s_), has to be determined, e.g., by comparison of the areas of corresponding tissue profiles of the fixed tissue sample (i.e., before embedding) and of the final histological section (i.e., after embedding), and correspondingly taken into account in calculation of (tissue-shrinkage sensitive) quantitative stereological parameters [1, 6]. Moreover, due to the harder consistency of polymerized plastic embedding media, thinner sections with lower inter-section variability can be cut from plastic-tissue blocks, providing a better recognizability of morphological details, and allowing for numerical quantification of small tissue structures by generation of physical disectors with low disector heights. Paraffin-embedding generally leads to a non-uniform, differential, anisotropic, not exactly predictable and variable tissue shrinkage, often by more than 50% in volume [6]. In contrast, embedding of tissue samples in a homogeneous plastic matrix is associated with a much lesser degree of tissue deformation [6]. For GMA/MMA embedded perusion-fixed murine kidney tissue, for example, the linear tissue shrinkage factor is f_s_ = 0.91 ±0.02, and for Epon embedding f_s_ = 0.95 ±0.02 (referring to a three-dimensional volume reduction of 22%, and 14%, respectively) [1]. Regarding the determination of section thicknesses, plastic embedding has additional, particularly important advantages, as compared to paraffin sections. Paraffin sections display a very uneven surface, since the paraffin is removed from the tissue section during the processing of the section (warmth-incubation, deparaffinization, rehydration, staining and dehydration), leading to an irregular collapse of the vertical height of the mounted tissue section (i.e., shrinkage in the vertical z-axis). In plastic sections, in contrast, the embedding medium is not removed during the subsequent procession steps, and the sections display uniformly smooth surfaces and equal section thickness in areas with and without embedded tissue (Fig 2).

### Previous methods of histological section thickness determination

Some methods for determination of histological section thicknesses calculate the average thickness of sections from a series of a known number of consecutive sections by measuring the reduction of the length of the tissue block during the sectioning process at the microtome [6, 12]. Such methods however, are prone to errors and can at most provide an approximate value of the average section thickness. The thicknesses of thick histological sections (<10 to >100 μm) can be determined directly using microscope systems equipped with mechanical or piezo-electrical z-axis steppers/measuring instruments, either by manual location of the upper and lower tissue surfaces within a section, or by using an automated absolute gradient focus function [13]. However, these methods of section thickness determination are generally not applicable for physical disector analyses, where usually thin sections (0.5–3 μm) are examined.

**Fig 3:**
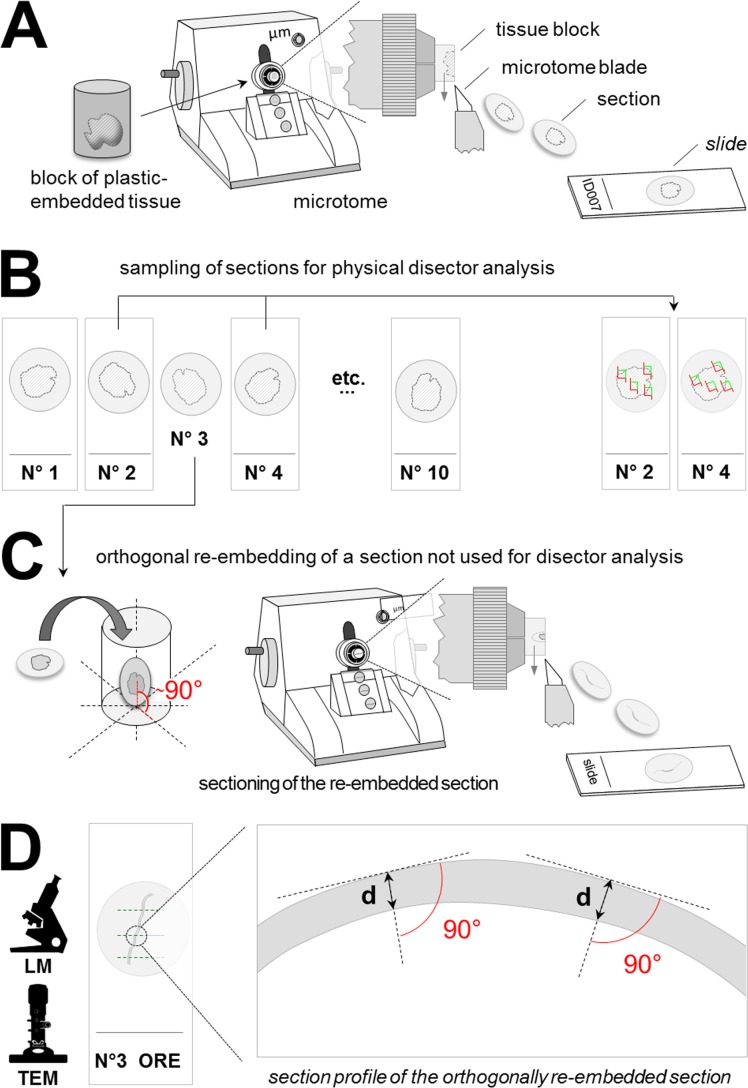
Schematic illustration of section thickness determination of orthogonally re-embedded sections. (A) A block of plastic-embedded tissue (e.g., Epon or GMA/MMA) is serially sectioned on a microtome. (B) From the series of consecutive sections (here N°1–10), section pairs are sampled for physical disector analysis (here sections N° 2 and N°4). C: From the remaining sections of the series, one section not used for disector analysis is sampled (here N°3) and re-embedded in plastic-embedding medium, vertically to its original section plane. The block with the re-embedded section is then sectioned with a microtome (for light microscopy, respectively with an ultra-microtome for electron microscopic examination). (D) The thickness of the orthogonally re-embedded section (d) is measured at randomly sampled locations, as the direct (orthogonal) distance between the upper and the lower cut-border of the section profile of the orthogonally re-embedded section, using light- (LM) or transmission electron-microscopy (TEM).

**Fig 4:**
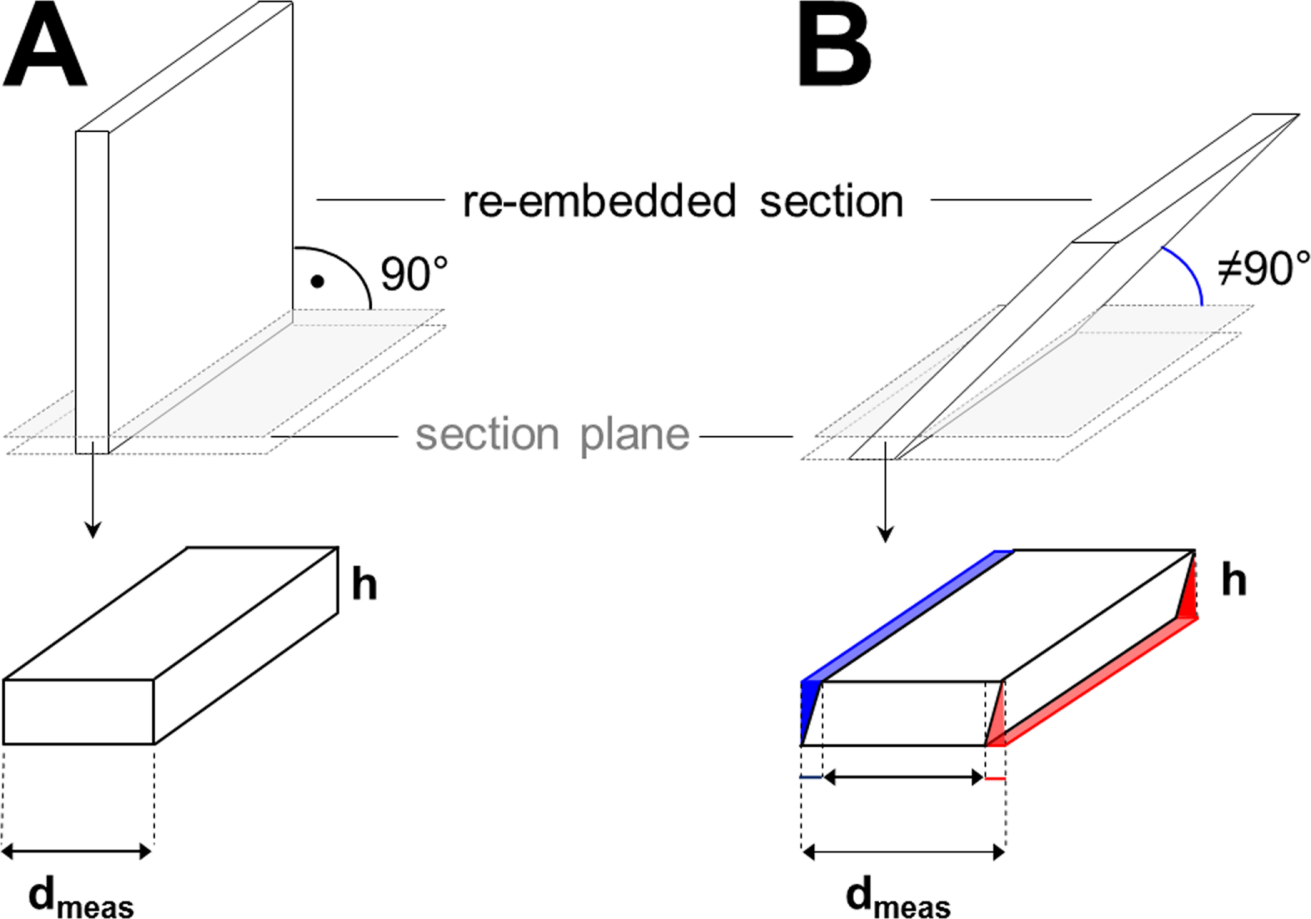
Effect of over-projection in determination of section thicknesses using vertically re-embedded sections. Two sections with the equal factual thickness d are re-embedded. (A) Ideal orthogonal re-embedding. The re-embedded section is re-sectioned exactly at 90° to its original section plane. The thickness h of the section of the re-embedded section does not affect the measured thickness d_meas_ of the re-embedded section, and d_meas_ is equal to the factual thickness of the section d. (B) Non-orthogonal (oblique) re-embedding. The re-embedded section is obliquely (≠ 90°) re-sectioned to its original section plane. Here, the measured thickness (d_meas_) of the (obliquely) re-embedded section exceeds the true thickness (d) of the re-embedded section S. This effect results from to the oblique sectioning angle (indicated in blue) and increases with the degree of deviation from the 90° angle and also from overprojection (indicated in red), increasing with the thickness of the sections cut from the obliquely re-embedded section. However, if GMA/MMA- or Epon sections are approximately vertically re-embedded in Epon and re-sectioned to 70–90 nm thin ultra-thin sections, the effect of overprojection in these ultra-thin sections will only marginally affect the measured thickness of the orthogonally re-embedded sections.

A few more methods have also been described for determination of the thicknesses of individual light- and electron-microscopic histological sections [13–17], as used in physical disector analyses in quantitative morphological studies. As a prerequisite for calculation of accurate and unbiased estimates of numerical volume densities in physical disector analyses, the thicknesses of individual (plastic) sections can precisely be determined, using the “orthogonal re-embedding (ORE) technique” (Fig 3) [1, 14, 16, 17]. From a series of consecutive sections, one (or more) section(s) not used for disector analyses is sampled and re-embedded in a plastic resin embedding medium, vertically to its original section plane. The thickness of the re-embedded section is determined in a subsequently prepared section of the plastic-block with the orthogonally re-embedded sample, by measuring the orthogonal distance between the upper and the lower surface of the section profile of the re-embedded section (Fig 3D). Due to the usually small nominal thickness (0.5–3 μm) of the re-embedded section, a precise measurement of its factual thickness in a 2D-section of the orthogonally re-embedded sample requires high factors of magnification. Therefore, these measurements are performed either using immersion-oil light microscopy (at 630–1000 x magnification) or transmission electron microscopy, allowing for application of higher magnification factors, thereby increasing the measurement’s accuracy. Moreover, since ultra-thin sections (approximately 70 nm thick) are used in transmission electron microscopic examination, the overprojection-effect of not completely vertically embedded sections is also minimized (Fig 4). However, orthogonal re-embedding of sections is labor-, time-, and cost-intensive, and it might produce inaccurate section thickness measurement values due to unintentional, oblique (non-orthogonal) positioning of the re-embedded sample-section.

Actually, the effect of accidental oblique embedding on the falsification of the measured section thicknesses might be considerable. If a section with a true thickness of 0.5 μm is re-sectioned with a deviation of, e.g., 35° from its original vertical plane, it will have a measured section thickness of 0.6 μm (i.e., 20% deviation from the true value), and if re-sectioned at an deviation of 45°, even 0.7 μm (i.e., 40% deviation from the true value).

#### An improved method for section thickness determination of orthogonally re-embedded sections

**Fig 5:**
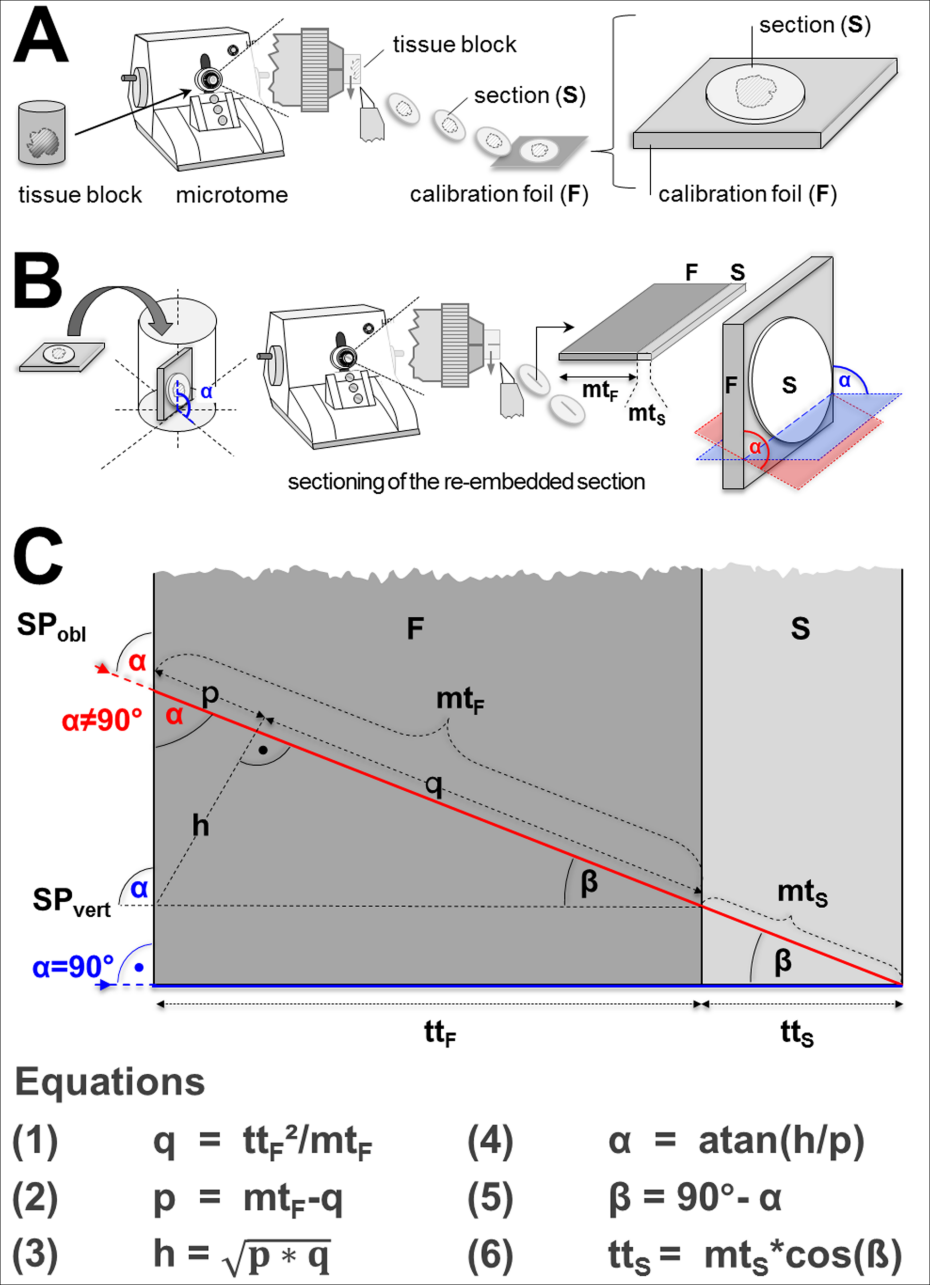
Schematic illustration of section thickness determination of orthogonally re-embedded sections and correction for non-vertical embedding. Compare to Fig 3. (A) A block of plastic-embedded tissue (e.g., Epon or GMA/MMA) is serially sectioned on a microtome. A section (S) sampled for determination of the section thickness is mounted flat on a calibration foil (F) of known thickness (tt_F_). (B) The section-calibration foil stack is re-embedded in plastic-embedding medium, vertically to the original section plane. The block with the re-embedded section and calibration foil is then sectioned with a microtome (for light microscopy, respectively with an ultra-microtome for electron microscopic examination). In the section of the section-calibration foil stack, the thickness of the orthogonally re-embedded section (mt_S_) is measured as the direct (orthogonal) distance between the upper and the lower cut-border of the section profile of the orthogonally re-embedded section. The thickness of the orthogonally re-embedded calibration foil (mt_F_) is measured accordingly. Depending on the angle (α) of the section plane relative to the level of the re-embedded section/calibration foil stack, the measured thicknesses of the section and the calibration foil exceed the true thicknesses of the section (tt_S_, unknown) and the foil (tt_F_, known). The blue section plane (SP_vert_) indicates an orthogonal section plane (α = 90°), the red section plane (SP_obl_) is cut at an oblique angle (α≠90°). C: The true factual thickness of the section (tt_S_) can be calculated geometrically, using the measured thicknesses of the calibration foil (mt_F_), the re-embedded section (mt_S_), and the known true thickness of the calibration foil (tt_F_). The mathematical equations (1–5) used for calculation of the angles and distances used for calculation of tt_S_ are displayed.

In the present report, a simple method is presented, allowing for determination of the factual section plane angle of the re-embedded section, and correction of measured section thickness values for an accidental/unintended oblique (non-orthogonal) position of section plane of the re-embedded section. The section that is to be orthogonally re-embedded is mounted flat on a calibration foil of known thickness. The section and the calibration foil are then vertically re- embedded and sectioned together. The section angle of the re-embedded section is then calculated from the deviation of the measured section thickness of the calibration foil and its factual thickness, using basic geometry (Fig 5).

Even though the effect of unintentional oblique embedding in ORE for section thickness determination can be compensated by using the method described here, ORE still remains quite time-, labor-, and cost-intensive. Moreover, since the determination of section thicknesses by ORE can usually not be performed on the identical sections that are also used in the disector analyses, (but on single sections of the same section series which are not used for quantitative stereological analysis, Figs 3B and 5A), the transferability of the measured section thicknesses on the other sections of the series that are used for disector analyses relies on the repetitive accuracy of the microtome in cutting section series with equal individual section thicknesses.

### Section thickness determination by spectral reflectance measurement

In order to overcome these problems, the present study also tested the suitability of a commercially available optical thin-film measurement device (F20, Filmetrics^®^) for quick and accurate determination of plastic section-thickness (GMA/MMA, Epon) by spectral reflectance measurement. Spectral reflectance measurement provides a fast, precise, and nondestructive tool, routinely used for a broad range of high-technology applications such as determination of the thicknesses of coatings of circuit boards and optical devices (color filters, high reflectivity mirrors, polarizers), or lacquer layers, as well as for determination of coating homogeneity, and roughness, or optical material constants [18]. While the theoretical physical foundations and technical details of the method are described elsewhere [19, 20], spectral reflectance (SR) measurement is principally based on analysis of the pattern of reflection(s) that occur at interfaces (i.e., top and bottom surfaces) of flat layers of different homogenous materials (film and substrate, such as e.g., a thin plastic section mounted on a glass slide), when light of different wavelengths is sent through such a “thin-film” stack. Due to the wavelike nature of light, light reflections at the partially reflecting surfaces of the different layers of the thin film will have different optical path lengths to pass through, and will, depending upon their wave-length and phase relationship, interfere with each other, resulting in detection of a particular interference pattern (i.e., a plot of the detected reflectance as a function of wavelength). Depending on the thicknesses of the materials of the thin film, their refractive indices and extinction coefficients (known material constants), and the incidence angle of the transmitted light (defined by construction), the detected spectral reflectance pattern displays characteristic intensity oscillations, whose amplitude and period can be used to determine the single film layer thicknesses.

The present report shows that, once the SR-measurement settings are defined for the different materials (i.e., the refractive indices, extinction coefficients, and approximate thicknesses of Epon-, and GMA/MMA sections and borosilicate-glass slides), SR can be used for convenient and reliable determination of the exact thicknesses of histological plastic sections within only a few seconds.

## Materials and methods

### Experimental setup

**Fig 6:**
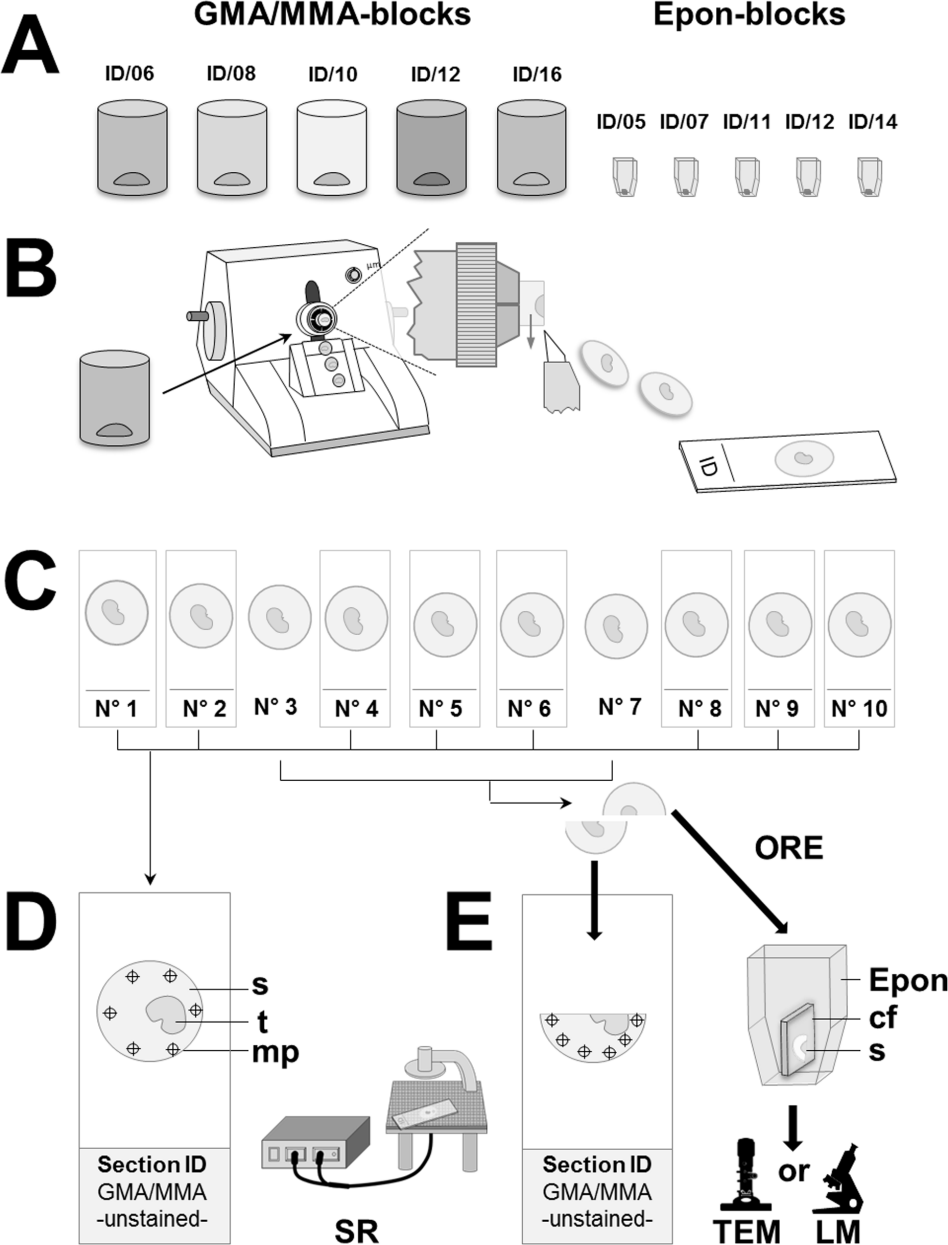
Experimental study design. Thicknesses of GMA/MMA- and of Epon sections were determined by spectral reflectance measurement and by light- and/or electron-microscopic measurement of the thicknesses of orthogonally re-embedded sections. (A) Sections were cut from five GMA/MMA-blocks and five Epon-blocks containing perfusion-fixed murine kidney tissue, (archive material) from 2005–2016 (different, independent casts). (B, C) From each GMA/MMA block, three series of each 10 consecutive sections were cut at nominal section thicknesses of 1, 2, and 3 μm. From each Epon block, a series of 10 sections with a nominal section thicknesses of 0.5 μm was cut (in C, only one section series of a GMA/MMA-block is shown). From each section series, 2–3 sections were randomly sampled (here: N°3 and N°7) for section thickness determination by orthogonal re-embedding (ORE) in Epon. GMA/MMA sections sampled for ORE were halved, whereas sampled Epon sections were completely re-embedded (not halved). The remaining sections were mounted on glass slides. (D) Per case, six spectral reflectance measurements (SR) of the section thickness were performed at measuring points/locations (mp) of the section (s), where no embedded tissue (t) was present. (E) From the halved GMA/MMA sections, each one half was mounted on a glass slide for spectral reflectance section thickness measurements, while the second section halves (s) were mounted flat on calibration foils (cf) and orthogonally re-embedded (ORE) in Epon and re-sectioned for verification of the section thickness by light microscopy (LM) of HE- or toluidin-blue stained sections. The thicknesses of orthogonally re-embedded Epon sections were determined by light- and by transmission electron-microscopic (TEM) measurements.

The suitability and the accuracy of spectral reflectance (SR) measurement for GMA/MMA-, and Epon section thickness determination was experimentally validated by comparison of section thicknesses assessed by SR measurements with light- and electron-microscopic measurements of the thicknesses of orthogonally re-embedded (ORE) sections. The experimental design of the study described in the following paragraphs is schematically outlined in Fig 6.

**Fig 7:**
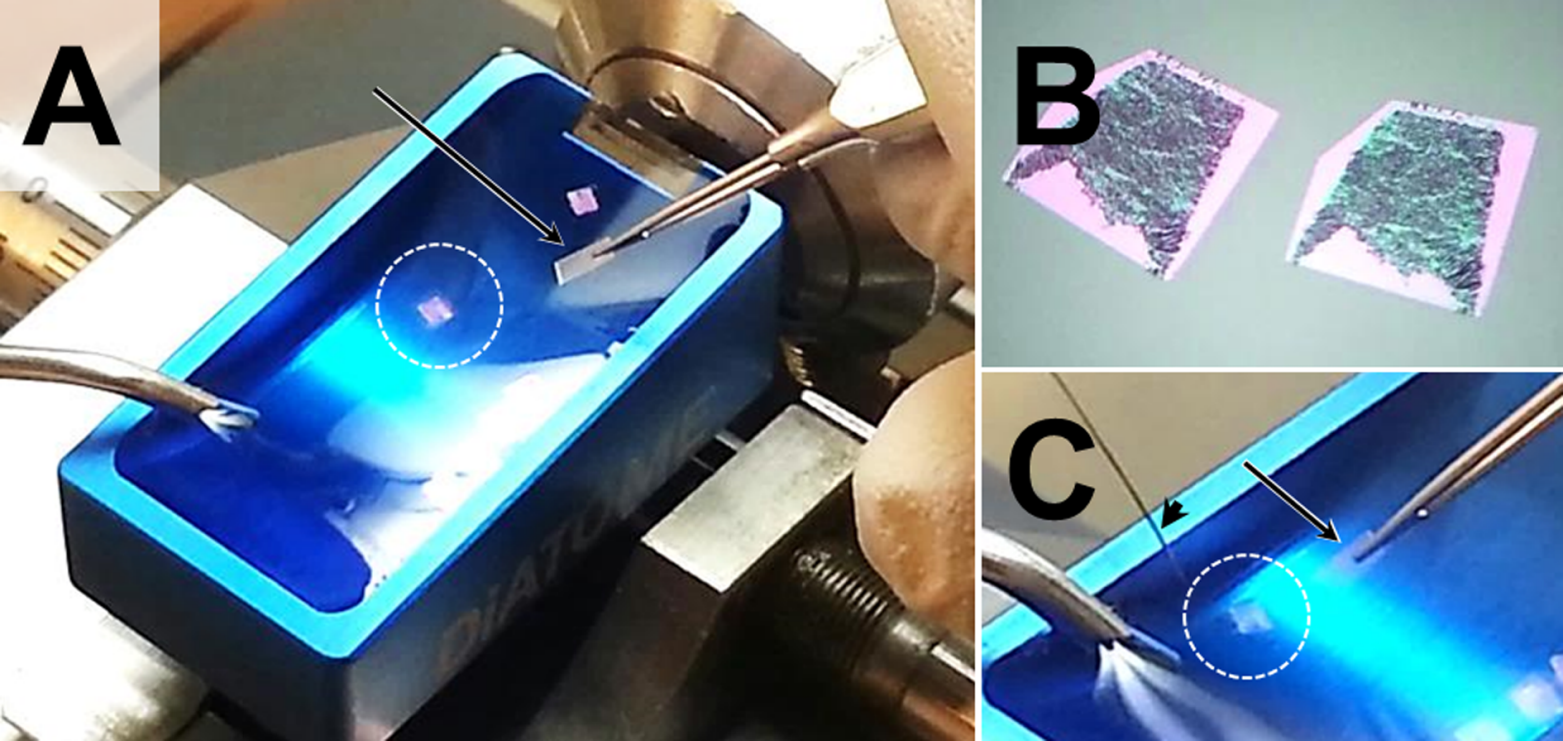
Mounting of sections (here: Epon sections) on calibration foils for subsequent orthogonal re-embedding. (A) Freshly sectioned Epon sections (encircled by a white dotted line) floating in the water bath (blue collecting basin) of the ultra-microtome. (B) Detail enlargement of Epon sections demonstrating a pink interference color. The grey-black material in the center of the section is the embedded tissue. (A, C) A section is carefully transferred to a stripe of calibration foil (arrow), using a horse hair (arrowhead in C).

Series of each 10 consecutive sections were cut from five blocks of GMA/MMA-, respectively of Epon embedded, perfusion-fixed (4% formaldehyde-solution) murine kidney tissue, prepared between 2006 and 2016 (archive material from different studies, Institute of Veterinary Pathology, LMU Munich, Germany). From each GMA/MMA block, three section series were cut at 1 μm, 2 μm, and 3 μm of nominal thickness, using a Microm HM 360 rotary microtome (Microm, Germany). An Ultracut E microtome (Leica, Germany) was used to cut a section series with 0.5 μm nominal thickness from each Epon block, with all sections of the series uniformly displaying a pink interference color (indicating uniform section thicknesses of individual sections, Fig 7B). From each section series, 2–3 sections were randomly sampled for section thickness determination by orthogonal re-embedding. GMA/MMA sections sampled for ORE were divided into two pieces. Each one half was mounted on an uncoated, standard, borosilicate glass slide (ISO 8037/1, Engelbrecht Medizin und Labortechnik GmbH, Edermünde, Germany) for SR section thickness measurement (see below), while the second section halves were mounted flat on calibration foils, orthogonally re-embedded in Epon using standard flat-embedding molds (in order to minimize the probability of unintended oblique (i.e., non-orthogonal) orientations of the section-calibration foil stacks within the Epon-blocks), and re-sectioned for verification of the section thickness by light-microscopic measurement (Fig 6E). Epon sections sampled for ORE were completely (not halved) mounted on calibration foil(s) and orthogonally re-embedded in Epon. The remaining sections of each section series (and the halves of GMA/MMA sections which were not orthogonally re-embedded) were mounted on uncoated, standard, borosilicate glass slides for SR measurement of section thicknesses, thereby also allowing for evaluation of section thickness uniformity within a series of consecutive sections.

### Orthogonal re-embedding of sections mounted on calibration foils

For light microscopic determination of the thickness of the orthogonally re-embedded sections, an ACLAR^®^ foil (Plano GmbH, Germany) with a nominal thickness of 198 μm was used as ORE-calibration foil in the present study. The ACLAR^®^ foil is a crystal clear, flexible, biochemically inert, heat-resistant, water-impermeable fluoropolymer film, widely used in electron microscopy. It displays only minimal dimensional change during embedding (<2%) [21]. Prior to ORE of sections, the thickness of the ACLAR^®^ foil was controlled by light microscopic measurement at 400x magnification, using an object micrometer (Zeiss, Germany) for calibration and accounted for 198.2 ± 1.8 μm on the average (78 single measurements at different locations).

For electron-microscopic determination of the thickness of the orthogonally re-embedded Epon- and GMA/MMA sections, calibration foils with certified thicknesses of 32 ± 1 μm and of 49 ± 1 μm (Calibration films, Art. 1120, LIST-MAGETIK^®^ GmbH, Leinfelden-Echterdingen, Germany), respectively of 12.46 ± 0.11 μm and 24.54 ± 0.4 μm (CPS-100 Certified Shim Set, serial N°: SCU100-0025, Check LINE^®^ Europe GmbH & Co KG, Gronau, Germany) were used (for electron microscopic section thickness determination of ORE sections, thinner calibration foils had to be used, since the diameter of the field of view in the smallest possible magnification factor (x1000) of the Zeiss EM-10 electron microscope is smaller than the thickness of the ACLAR^®^ foil of ~200 μm). The used LIST-MAGETIK^®^ and Check LINE^®^ calibration films, are made of a flexible plastic foil, with advantageous chemical-physical properties similar to that of the ACLAR^®^ foil (flexible, easily sectionable, resistant to water, alcohol, aceton).

GMA/MMA-, respectively Epon sections mounted on calibration foils were orthogonally re-embedded in Epon, using standard flat-embedding molds. Subsequently, semi-thin (approximately 0.5 μm thick) Epon sections of orthogonally re-embedded GMA/MMA- and Epon sections and calibration foils were prepared, mounted on glass slides (ISO 8037/1, Engelbrecht Medizin und Labortechnik GmbH, Edermünde, Germany) and stained with HE (sections of re-embedded, nominal 1, 2, and 3 μm thick GMA/MMA sections) or toluidin-blue (sections of re-embedded, nominal 0.5 μm thick Epon sections).

**Fig 8:**
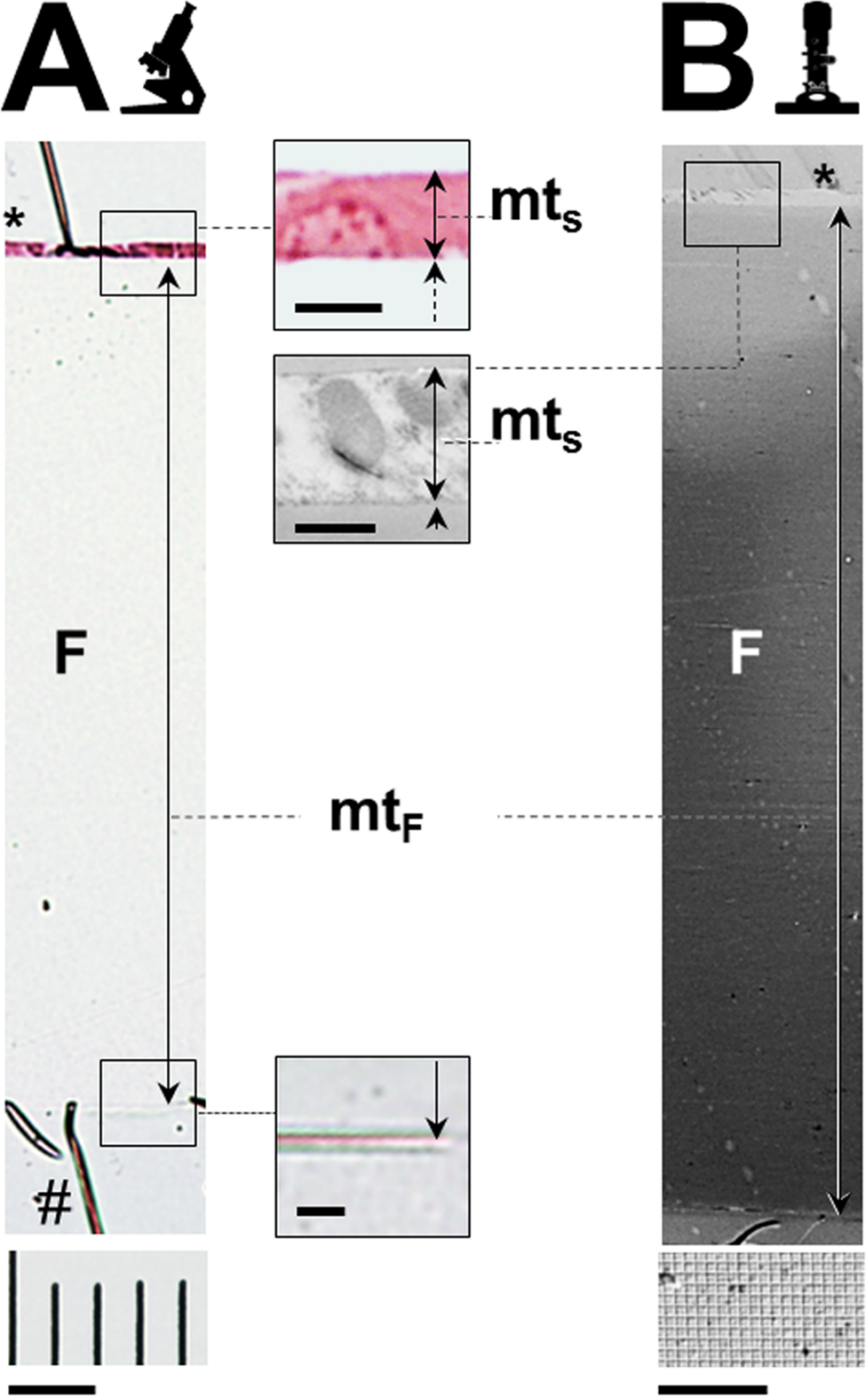
Light- and electron-microscopic images of sections of re-embedded GMA/MMA sections mounted on calibration foils. Compare to Fig 3. The thicknesses (mt_F_) of the calibration foils (F) and the thicknesses (mt_S_) of the re-embedded sections (*) are indicated. (A) Light microscopic image of a re-embedded, 3 μm thick GMA/MMA section of mouse kidney tissue mounted on a calibration foil (ACLAR^®^, Plano, Germany) of 198 μm (true) thickness. #: Tension fold at the interface of the calibration foil and the surrounding Epon resin. GMA/MMA section, HE-staining, 200 x magnification. For calibration, the image of an object micrometer (distance between scale lines: 10 μm) photographed under identical conditions is displayed (bar = 20 μm). The insets to A show detail enlargements of the profile of the orthogonally re-embedded tissue section (top inset, bar = 5 μm) and the lower surface of the calibration foil (bottom inset, bar = 5 μm). (B) Transmission electron microscopic image of a re-embedded, 1 μm thick GMA/MMA section of mouse kidney tissue mounted on a calibration foil (LIST-MAGETIK^®^, Germany) of 49 μm (true) thickness. 1000 x magnification. For calibration, the image of a standard cross-grating calibration grid (width of squares: 0.463 μm) photographed under identical conditions is displayed (bar = 10 squares). The inset to B shows a detail enlargement of the profile of the orthogonally re-embedded tissue section (10,000 x magnification, bar = 0.463 μm).

Light microscopic measurements of the thicknesses of the orthogonally re-embedded sections were performed at a 200 x magnification (measurement of the calibration foil thickness), respectively at 1000 x (oil immersion) magnification (measurement of the section thickness), using an automated stereology system (VIS-Visiopharm Integrator System^TM^ Version 3.4.1.0 with newCAST^TM^ software, Visiopharm A/S, Denmark). The thicknesses of the calibration foil and the overlying section were determined at 6 locations (approx. 150 μm apart, with a random position of the first measurement location) per case, measuring the shortest (orthogonal) distance from the lower to the upper surface of the calibration foil and the corresponding distance between the lower and the upper surface of the overlying section at the same location. Morphometric section thickness measurements were only conducted in areas, where no folds were present, and were the orthogonally re-embedded section was in direct, flush-even (plan) contact with the calibration foil (Fig 8A).

The locations where section thickness measurements of orthogonally re-embedded sections were performed included positions where embedded tissue was present in the section, as well as positions without embedded tissue (to confirm the uniform thickness of areas with and without embedded tissue in the same section, the thicknesses of orthogonally re-embedded GMA/MMA- and Epon sections were additionally measured in each 3 locations with-, and in 3 locations without embedded tissue per case and compared).

The measured section- and calibration-foil thickness values were averaged per case and the true thickness of the orthogonally re-embedded section (i.e., corrected for unintentional oblique embedding/sectioning) was calculated as described above.

For electron microscopic measurement of the thickness of orthogonally re-embedded Epon sections of 0.5 μm nominal thickness, ultra-thin sections (~70 nm) were cut from the corresponding Epon-blocks, using an Ultracut E microtome (Leica, Germany), routinely processed for electron microscopy, and examined using a Zeiss EM-10 transmission electron microscope (Zeiss, Germany). Digital micrographs of the orthogonally re-embedded sections, as well as of a standard cross-grating calibration grid (S107, TAB, USA) were taken at 1000–10.000 x magnification (Fig 8B). Section thickness measurements were performed at three to six locations per case (approximately 10–50 μm apart), and the measured values were averaged per case (section).

### Scanning electron microscopy

For demonstration of the ultrastructure of the section surfaces of paraffin- and plastic sections (Fig 2), scanning electron microscopy was performed following standard protocols, using a digital scanning electron microscope (Zeiss DSM 950, Carl Zeiss AG, Germany).

### Spectral reflectance measurements

**Fig 9:**
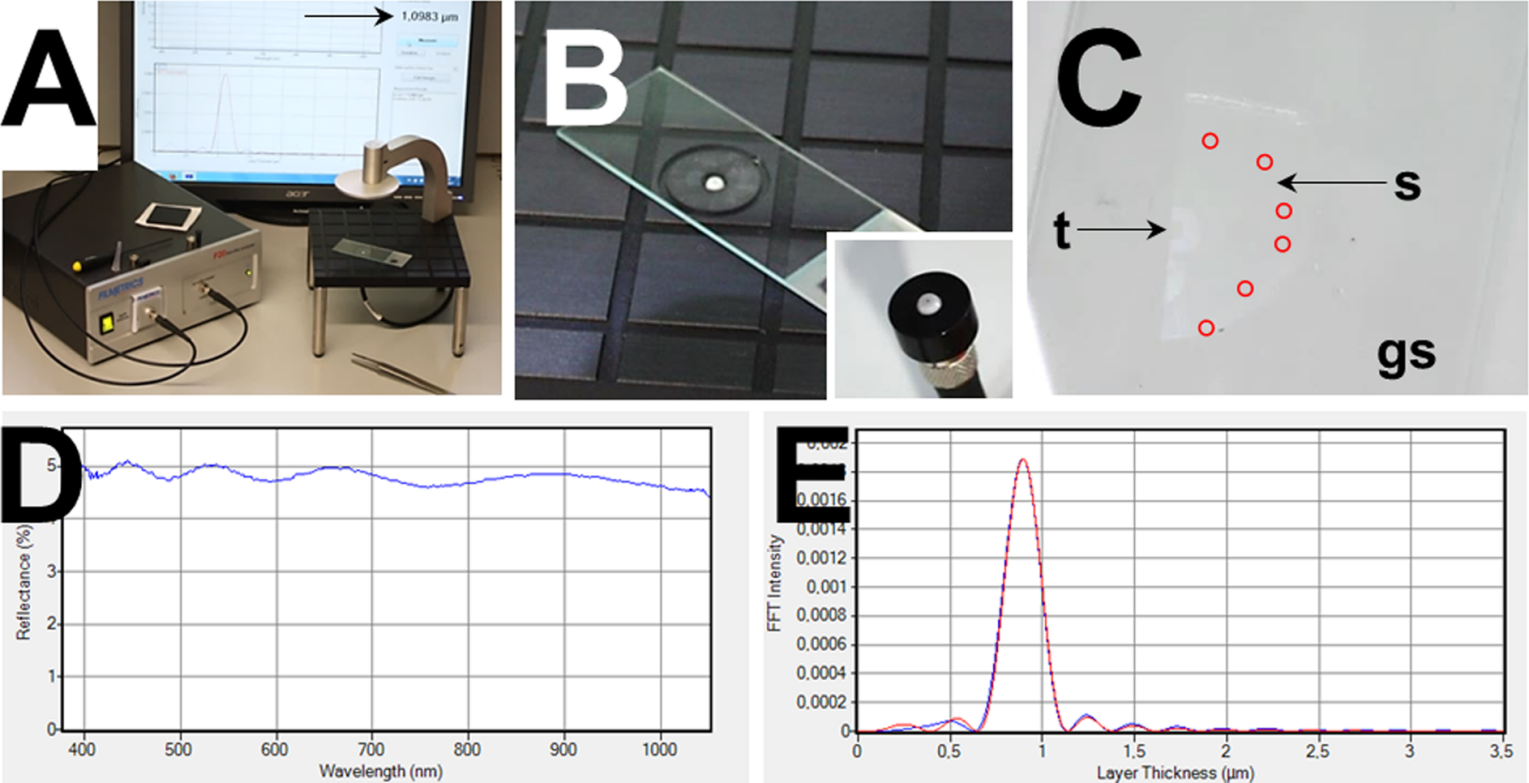
Spectral reflectance section thickness measurement. Thicknesses of unstained GMA/MMA or Epon sections mounted on borosilicate glass slides were measured with a F20 optical reflectometer (Filmetrics®, USA) using the “contact stage” mode. The glass slide is placed on the stage with the mounted section facing the opening of the fiber optic cable (A, B). The opening of the fiber optic cable (inset to B) has a diameter of ~250 μm. (C) Detail enlargement of one half of a GMA/MMA section (s, arrow) mounted on a glass slide (gs) for spectral reflectance measurement of the section thickness (the second half is orthogonally re-embedded in Epon for verification of the section thickness by microscopic measurement). The tissue (t) present in the section is indicated (arrow). Per case, section thickness measurements were performed at six different locations (indicated by red circles) of the section, where no embedded tissue was present. Measurement details (D, E) and results are directly displayed at the monitor of the connected computer (A, arrow). (D) Reflectance spectrum. (E) Fast Fourier Transform (FFT)-intensity plot. The blue line on the graph represents the measured reflectance data, whereas the red line on the graph shows the calculated reflectance (based on the indicated refractive indices, extinction coefficients, and approximate thicknesses of the Epon-, or GMA/MMA sections and the borosilicate-glass slides). A successful measurement is indicated by an overlap of the wavelengths of the maxima and the minima of the calculated and the measured reflectance curve.

The optical reflectometer (F20, Filmetrics^®^) used in the present study consists of a housing containing the light source (tungsten halogen bulb, generating light from approximately 375–3000 nm wavelength) and spectrometer (detector), a fiber optic cable and a stage for positioning of the light outlet (Ø ~ 0.25 mm) of the fiber optic cable and the sample during measurement (Fig 9). The device is connected to a computer with the appropriate software installed. Measurement details (i.e., acquired reflectance spectra) for evaluation of the measurement’s quality and the result each single measurement are immediately displayed (a single measurement is completed within 1 second) and automatically secured for subsequent further analysis.

For thickness measurement of GMA/MMA and Epon sections, the “Contact Stage” mode (Figs 6E, 9A and 9B) was used. Measurement parameters and sample specifications were set according to the FILMETRICS F20 Operations Manual (2016, Revision 7.17.6.0) as specified in Table 2.

**Table 2:** Measurement settings and sample specifications for thickness measurement of GMA/MMA and Epon sections with the F20 optical reflectometer (Filmetrics^®^, USA).

Measure–Film recipe		Units	Microns (μm)
Film stack	Medium	Air	
**Layers**	**1**	**GMA/MMA** sections	**Acrylic** (predefined)
			**-Grading: 0%**
			**-Thickness:**
			**-Nominal (μm): 2**
			**-Range (μm): ± 1.5, tick box**
			**-Refine via: None**
		**Epon** sections	**Generic, n = 1.491** [22]
			**-Grading: 0%**
			**-Thickness:**
			**-Nominal (μm): 0.5**
			**-Range (μm): ± 0.15, tick box**
			**-Refine via: None**
	**Substrate**	**Borosilicate glass (BSG)**	
**Analysis options**		Wavelength Range:	
		-Fixed range-From/To: Default-value (nm)	
		Smoothing Optical thickness: 60 μm	
		Source Data:	
		-Analyze using Reflectance 0°, tick box	
		**-Analysis method: FFT**^a^ **(thickness only), tick box**	
	Advanced	FFT Analysis Window:	
		**-No optimization, tick box**	
		-FFT Total Thickness: Min. Peak Height: 50%	
**Alarms**		**GMA/MMA** sections	Minimum valid GOF: 0
			Layer 1
			Nominal: 2, Min: 0, Max: 1.5
			Minimum valid GOF: 0
		**Epon** sections	Layer 1
			Nominal: 0.5, Min: 0, Max: 1.6
**Acquisition**		Measurement Timing	
		-Use recommended sampling time: Unknown (ms)	
		Optics: Contact stage, tick box	
	Advanced	Store Baseline in Recipe, tick box	
		**Optics Configuration: Contact stage**	
		-Baseline name: Default	
		**-Reflectance Standard: BK7**	
	Optics	**Contact stage**, tick box (no Auto Baseline)	

^a^FFT: Fast Fourier Transform

Prior to, and in regular intervals during the measurements, “Baseline” calibrations of the system were performed using the provided BK7 reflectance standard. For measurement of section thicknesses, the glass slide with the mounted section (unstained histological section without a coverslip) was placed flat on the stage, in such a way that an area of the section not containing embedded tissue was located directly above the small light source opening of the fiber optic cable.

In each single section, four to six spectral reflectance measurements of the section thickness were performed at locations, where no embedded tissue was present (Fig 9C). For comparison, measurements were also performed in areas of GMA/MMA sections where identifiable tissue was present (each six measurements per case). Directly after completion of each measurement, the result (calculated section thickness) and analysis details (reflectance- and Fast Fourier Transform (FFT)-intensity spectra) were displayed. The quality of each measurement was estimated by evaluation of the shapes of the reflectance- and FFT-intensity plot curves and the congruence of estimated and measured FFT-intensities (Fig 9D and 9E).

### Statistical analyses

Data are presented as means ± standard deviations (SD). Statistical analyses of the deviations of section thickness measurement values determined by spectral reflectance measurement and orthogonal re-embedding of sections were performed, using two-sided, paired student’s t-tests (Microsoft EXCEL^®^). P values <0.05 were considered significant.

## Results and discussion

### Orthogonal re-embedding of sections mounted on calibration foils

Epon- and GMA/MMA sections adhered well to the ACLAR^®^ foil, as well as to the LIST-MAGETIK^®^, and the Check LINE^®^ calibration films. The calibration foils with mounted GMA/MMA- and Epon sections could subsequently be embedded in Epon, and, after polymerization, sectioned without greater difficulties. In some of the orthogonally re-embedded sections, small tension folds were present at the interfaces of the calibration foil and the surrounding Epon resin (Fig 8A#), and in few locations, the orthogonally re-embedded-sections were also slightly detached from the calibration foil. Therefore, morphometric section thickness measurements were only conducted in areas, where no such folds were present, and were the orthogonally re-embedded section was in direct, flush-even (flat) contact with the calibration foil (Fig 8).

Within the same section, the thicknesses of areas with and without embedded tissue were virtually equal (p >0.05). On the average, the deviation of the thicknesses of section areas with and without embedded tissue were 1.3 ± 1.4%, -1.5 ± 1.7%, and -1.9 ± 1.6% for GMA/MMA sections of 1, 2, and 3 μm nominal thickness, respectively, and 0.5 ± 0.9% for Epon sections of 0.5 μm nominal thickness.

The effect of oblique re-embedding on the measured thicknesses of sections could be corrected as described above, using the measured- and the known true thicknesses of the co-sectioned calibration foil. Of note, using flat-embedding molds for orthogonal re-embedding of section-calibration foil stacks usually prevented severe unintended oblique embedding angles. In the present study, the oblique embedding angles (ß), determined as described above, ranged from 3° to 15° (10 ± 2°), referring to an average deviation of the measured and the true section thicknesses of 2 ± 2% (range: 0.2% to 3.7%).

### Spectral reflectance measurements

Using the Filmetrics^®^ F20 reflectometer as described above, section thickness measurements were performed within approximately one second per measurement and the quality of each single measurement could immediately be evaluated. The more than 1000 single measurements of the present study were performed within less than 4 hours, without any haste. All individual spectral reflectance measurement data of the present study are provided as supporting information (S1 File). Whereas measurements carried out at locations of the section where no embedded tissue was present consistently produced reflectance spectra and FFT-intensity plot curves indicating good data qualities, measurements performed in locations where tissue was present in the section (especially in sections of > 2 μm nominal section thickness) regularly yielded lower (on the average 16 ± 16%) section thickness measurement values with up to 3.5 times higher inter-measurement variabilities and analysis spectra indicating measurement results of limited reliability (S1 File). The observed ineffectiveness of spectral reflectance measurement for section thickness determination in section areas containing embedded tissue is likely a consequence of the physical operating principle of the method, since the presence of embedded tissue will introduce additional, heterogeneous, and irregularly oriented light-reflecting tissue-plastic interfaces within the plastic section. Therefore, plastic section thickness measurements by spectral reflectance should only be performed in “empty” section areas not containing embedded tissue.

The mean deviation of the 4–6 single section thickness measurement values determined by spectral reflectance measurement in each examined section was 0.06 ± 0.05 μm in GMA/MMA sections of 1–3 μm nominal section thickness, and 0.007 ± 0.008 μm in Epon sections of 0.5 μm nominal section thickness. Besides, characterizing the repetitive accuracy of the microtomes used in this study, the thicknesses of individual sections of single series of consecutive sections (sectioned from an individual GMA/MMA- or Epon block with a defined nominal section thickness) determined by spectral reflectance measurement displayed only little inter-section variability (Table 3).

**Table 3:** Mean deviation of spectral reflectance section thickness measurement values in series of (consecutive) plastic sections (inter-section variability of section thickness).

Embedding medium	Nominal section thickness (μm) of series	Mean deviation of single measured section thickness values per section series			
		Mean (μm)	SD (μm)	% of mean	SD (%)
**GMA/MMA**	**1**	0.09	± 0.02	9.3	± 1.8
	**2**	0.09	± 0.04	4.9	± 2.0
	**3**	0.10	± 0.01	3.7	± 0.3
	**1–3**	0.09	± 0.02	6.0	± 2.9
**Epon**	**0.5**	0.017	± 0.004	3.7	± 1.0

Data are means of the mean deviations of measured individual section thickness values of the sections of different section series. Section thicknesses were determined by spectral reflectance measurements in 150 individual GMA/MMA sections from 5 section series with 1 μm, 2 μm, and 3 μm nominal section thickness and 35 individual Epon sections from 5 section series with 0.5 μm nominal section thickness.

On the average, the thicknesses of GMA/MMA-, respectively of Epon sections determined by spectral reflectance measurement and by light- and/or electron-microscopic measurement of orthogonally re-embedded sections differed only minimally (< 1%), with deviations ranging from -0.01 μm to 0.02 μm in Epon sections of 0.5 μm nominal thickness, respectively from -0.06 to 0.08 μm in GMA/MMA sections of 1–3 μm nominal thickness (Table 4).

**Table 4:** Deviation of section thickness measurement values determined by spectral reflectance measurement and orthogonal re-embedding of (identical) sections.

	Light microscopic measurement of ORE section thicknesses				Electron microscopic measurement of ORE section thicknesses	
Embedding medium	GMA/MMA*		Epon		Epon	
Deviation ORE-SR	Absolute (μm)	% of OTE	Absolute (μm)	% of OTE	Absolute (μm)	% of OTE
**Mean**	**0.01**	**0.6**	**0.001**	**0.3**	**-0.001**	**-0.13**
**SD**	±0.04	±2.3	±0.01	±2.4	±0.01	±1.75
**Min**	-0.06	-3.8	-0.01	-1.9	-0.01	-4.91
**Max**	0.08	3.7	0.01	3.1	0.02	2.82
**p**	0.28 (n.s.)	0.34 (n.s.)	0.88 (n.s.)	0.78 (n.s.)	0.93 (n.s.)	0.96 (n.s.)

Data are means of the deviations of section thickness measurement values determined by spectral reflectance measurement and orthogonal re-embedding of (identical) sections.

* For GMA/MMA sections, the averaged section thickness values of sections of 1, 2, and 3 μm nominal thickness are shown.

ORE: Measured thickness of orthogonally re-embedded section. SR: Section thickness measured by spectral reflectance. SD: Standard deviation. p: p-values of paired student’s t-tests.

In summary, these results demonstrate the suitability of spectral reflectance analysis for determination of thicknesses of plastic-sections. Compared to alternative methods, such as measurement of section thickness in sections of orthogonally re-embedded sections, spectral reflectance analysis has significant advantages. It is much faster, far less work-, and cost-intensive, and, due to its contact-free, non-destructive nature, it can be applied to the identical sections used for subsequent quantitative morphological analysis. The tested plastic-embedding media (Epon and GMA/MMA) and section thicknesses (0.5–3 μm) cover a broad range of possible applications in quantitative stereological studies and are regularly used in physical disector analyses of different tissues [1, 23, 24]. Here, spectral reflectance analysis proved to enable accurate and reliable, uncomplicated, fast and comparably inexpensive determination of plastic section thicknesses, predestining this method to be routinely performed in all types of quantitative stereological analyses estimating parameters depending on the thicknesses of the examined sections. If section thickness determination by spectral reflectance measurement should not be available, the improved method of orthogonal re-embedding with correction of unintended oblique sectioning described in the present study is recommended.

## Supporting information

S1 File Spectral reflectance measurement data. List of all individual spectral reflectance measurement values of section thickness measurements in the present study.
(XLSX)
